# Long‐distance gene flow in *Acacia senegal*: Hope for disturbed and fragmented populations

**DOI:** 10.1002/ece3.10292

**Published:** 2023-07-12

**Authors:** Stephen F. Omondi, Eunice W. Githae, Damase P. Khasa

**Affiliations:** ^1^ Department of Forest Genetics and Tree Improvement Kenya Forestry Research Institute Nairobi Kenya; ^2^ Department of Biological Sciences Chuka University Chuka Kenya; ^3^ Centre for Forest Research and Institute for Systems and Integrative Biology Université Laval Sainte‐Foy Québec Canada

**Keywords:** *Acacia senegal*, anthropogenic disturbance, gene dispersal, genetic structure, mating systems

## Abstract

Even though pollen and seed dispersals are some of the important factors that determine tree species survival across landscapes, gene dispersal data of important tropical dryland tree species such as *Acacia senegal* that are undergoing various population disturbances remain scarce. Understanding patterns of gene dispersal in these ecosystems is important for conservation, landscape restoration and tree improvement. We investigated pollen and seed mediated gene flow in two *A. senegal* populations of contrasting state (less disturbed and heavily undisturbed) using nine microsatellites and 128 genotyping‐by‐sequencing single nucleotide polymorphism (SNPs) multilocus genotypes of two growth stages (juvenile and adult trees) and their spatial locations. We performed parentage assignments using likelihood approach and undertook spatial genetic structure (SGS) analyses for the two growth stages through correlation among kinship coefficients and geographical distances between pair of individuals. The SNPs showed higher resolving power and assignment rates than microsatellites; however, a combination of the two marker‐types improved the assignment rate and provided robust parentage assessments. We found evidence of long‐distance (up to 210 m) pollination events for both populations; however, the majority of seed dispersal was found closer to the putative maternal parent. On average, parentage analysis showed high amounts of pollen (40%) and seed (20%) immigration in both populations. Significant positive SGS was found only for the adult cohorts in the less disturbed population for distance classes 20 and 40 m, indicating historical short‐distance seed dispersals. Our results suggest long‐distance gene flow within the species and we recommend conservation of remnant and isolated populations or individual trees to promote genetic connectivity.

## Graphical abstract

Understanding patterns of gene dispersal in tropical dryland species such as *Acacia senegal* is important for conservation, landscape restoration, and tree improvement program. We investigated pollen and seed mediated gene flow in two *A. senegal* populations of contrasting state (disturbed and undisturbed) using multilocus genotypes of seedlings and potential parent trees and their spatial locations. Our results suggest long‐distance gene flow within the species and recommend conservation of remnant and isolated populations or individual trees to promote genetic connectivity.

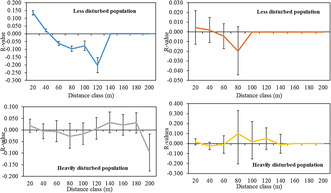

## 1 INTRODUCTION

Gene flow in plants is mediated by both pollen and seed dispersal, which vary greatly in terms of mechanism and extent among species (Kremer et al., 2012; Marchini et al., 2016). It is one of the main factors determining the genetic architecture of populations together with drift, selection, and mutation (Burczyk et al., 2004; Grasty et al., 2019). Pollen and seed dispersal, however, have distinct effects on the rate of demographic expansion, and the rate at which genes move across species range. In this light, pollen and seed movements are key parameters in shaping the dynamics and evolution of plant populations (Hardy, 2009). Overall, patterns of pollen and seed‐mediated gene flow and variation in the mating system directly influence levels of genetic diversity, connectivity, and population structure. These factors are essential to plant populations' evolutionary potential (Cristóbal‐Pérez et al., 2021; Eckert et al., 2010). Population genetics theory predicts disruption of genetic connectivity when populations become small and fragmented or geographically isolated (Klein et al., 2013). Furthermore, loss of allelic diversity via increased levels of genetic drift is anticipated to result in reduced levels of genetic diversity within populations and increased genetic divergence among populations (Aguilar et al., 2019). In many cases, the spatial scale of effective propagule dispersal in trees depends on a variety of physical and biological processes that determine the amount and availability of pollen and seeds. These include propagules movement, viability and the probability of successful pollination leading to viable seed development and seedling establishment rates (Kremer et al., 2012). Different combinations of these processes may yield effective dispersal distances spanning from a few meters to several kilometers, generally following markedly leptokurtic patterns (González‐Robles et al., 2021; Kremer et al., 2012).

Reduced plant population size and increased isolation through natural or anthropogenic disturbances may influence mating patterns, with reduced numbers of existing mates, increased levels of self‐fertilization, and reduced levels of reproductive success and adaptive fitness in progenies due to inbreeding depression (Jacquemyn et al., 2012). Historically, disturbed species habitats often have small effective population sizes, and geographically fragmented and patchily distributed populations with geographically restricted ranges (Burczyk et al., 2004; Solís‐Hernández & Fuchs, 2019). In agreement with population genetic theory, the influence of these factors on gene flow is expected to be largely negative (Cristóbal‐Pérez et al., 2021). Meta‐analyses have shown that species in disturbed habitats are generally associated with low overall genetic diversity and increased levels of among‐population genetic structure as a result of the heightened impacts of genetic drift under conditions of limited genetic connectivity and/or selection under a narrow range of environmental conditions (Leimu et al., 2006). The long‐term impacts of restricted gene flow on species of disturbed habitats with small fragmented populations and geographically restricted ranges may include risk of extinction (Ellstrand & Ellam, 1993). Accurate estimations of gene dispersal both by pollen and seed are essential for assessing correlates of fitness in natural populations and determining the role of sexual selection and ecological factors in breeding systems evolution (Burczyk et al., 2004; Cristóbal‐Pérez et al., 2021). This would be useful in designing reserves and conservation strategies that maximize populations' interconnectedness.

Anthropogenic disturbances and habitat fragmentation are quite common in the tropical landscape and represent the major threat for genetic diversity maintenance and natural plant populations' viability (Quesada et al., 2013; White et al., 2002). These threats can adversely affect the exchange of genetic information within remnant populations by promoting reproductive isolation through effective population size reduction and altered pollen and seed dispersal patterns (Solís‐Hernández & Fuchs, 2019). The impacts of disturbance on genetic structure and gene flow within fragmented forest landscapes are, however, poorly understood, especially in tropical dryland ecosystems (White et al., 2002). Through human disturbances, decline in population size may reduce reproductive individuals' density. Such disturbances may increase isolation and its effects on the underlying forces of gene flow, which may directly influence the genetic structure within a fragment (Aguilar et al., 2008). Simultaneously, spatial isolation between individuals and relict populations may restrict connectivity because of low levels of pollen and seed dispersal between patches and in the long term may cause loss of genetic variability in the remnant tree populations due to genetic drift and high levels of inbreeding (Sork & Smouse, 2006; Xiao et al., 2016).

*Acacia senegal* trees are highly valued for gum arabic production in the drylands of sub‐Saharan Africa (Chikamai & Odera, 2002). The gum is collected as natural or artificially tapped exudates from stem and branches and traded locally and internationally hence significantly contributing to the livelihoods of local populations and the general economy of the countries involved (Fagg & Allison, 2004; Wekesa et al., 2013). Additionally, the species is also useful in agroforestry and fuel wood production (Ballal et al., 2005). Distribution of the species is mainly in the drylands of Kenya; however, some populations are threatened by human disturbances (Omondi et al., 2016). Recent studies show that some *A. senegal* populations have suffered from selective harvesting and land clearance for agricultural production and settlements (Lelon, 2008; Omondi et al., 2016). Such disturbances may affect the species' biological processes including mating systems and gene flow patterns which may be detrimental to the species evolutionary potential and population stability (Omondi et al., 2016). However, no study has investigated the effects of human disturbances on gene flow patterns within the species. Such studies are essential components of designing conservation and restoration plans of degraded landscapes.

Studies show that tropical trees are particularly vulnerable to the effects of habitat disturbances and fragmentation because they naturally occur at low density and have specialized interactions with pollinators and seed dispersers (Ward et al., 2005). It has also been shown that habitat disturbances negatively affect plant reproduction by reducing pollinator activity, pollen deposition, fruit set, outcrossing rates, and the number of progenies in disturbed landscapes (Aguilar et al., 2006, 2008, 2019; Cunningham, 2000). However, empirical evidence shows contrasting results, in some species. Under certain conditions, habitat disturbance and fragmentation can improve gene movement and connectivity between isolated habitats which ultimately reduces genetic differentiation and maintains high levels of genetic diversity in the long‐term (Dick et al., 2003; Lowe et al., 2005). Such contrasting evidence may be brought about by the quality of dispersal of different gene vectors and the degree of isolation and the size of the remnant populations (Fuchs et al., 2003; Nason & Hamrick, 1997). Although pollen flow can persist in fragmented habitats, remnant forest patches may be at risk for loss of genetic diversity because of a reduced number of local and immigrant pollen sources (Sork & Smouse, 2006). Therefore, integration of estimated gene dispersal distance with data on the genetic diversity of pollen pools is required to understand whether isolation and reduction of population size due to anthropogenic disturbances and fragmentation have negative effects on plant reproduction and mating patterns of tree populations.

The aim of the present study was to investigate the patterns of gene flow within two natural populations of *A. senegal* with contrasting disturbance levels so as to determine the effect of habitat disturbance on gene flow patterns. We also evaluated the impact of gene flow on the spatial genetic structure (SGS) of the two populations. Our study hypothesize that the heavily disturbed population of *A. senegal* will display restricted gene flow pattern characterized by short‐distance pollen and seed dispersal, leading to non‐random distribution of genotypes in space compared to the less disturbed population. We used mating patterns and the SGSs of the pre (mature individuals)‐ and post (juvenile individuals)‐disturbance gene pools to illustrate the extent of gene flow, and separated them into components of identified local against unidentified putative local or immigrant genes. We achieved the aims of the present study using both microsatellite and single nucleotide polymorphism (SNP) markers. Implications of the study findings for conservation and management of the species are discussed.

## 2 MATERIALS AND METHODS

### 2.1 Study sites

Two natural populations of *A. senegal* within Lake Baringo woodland, Solit (00°14′N and 36°35′E) and Kimalel (00°21′N and 36°03′E), with contrasting anthropogenic disturbance status were studied. We surveyed the two populations prior to the study and classified them as heavily disturbed (Solit) or less disturbed (Kimalel) based on population disturbance index (PDI) and density of adult trees (Table 1) as reported in Omondi et al. (2016). The PDI comprised of presence/absence of seven disturbance indicator variables including; number of cut tree stumps (percentage proportion of cut stumps against the total number of mature stem), number of old and new charcoal kilns, presence of grazing livestock, level of seedlings browsing (percentage proportion of the number of browsed seedlings against the total number of seedlings), number of recent settlements, number of fenced plots, and presence of cultivated agricultural land (Table 1). For each population, the PDI was calculated as the sum of all present indicators. The choice of indicator variables used in this study was informed by existing literature on factor affecting vegetation dynamics within Lake Baringo ecosystem (Omondi et al., 2016 and references therein) and personal observations. Density of adult *A. senegal* trees was determined by performing inventory of 15 plots measuring 20 × 20 m (400 m^2^) per population. Mean number of adult trees (diameter at breast height, dbh > 5 cm) per hectare was determined for each population. In this study, a tree was considered to be in disturbed habitat when PDI of the population was greater than 5 (presence of more than 60% of disturbance indicators) and the density of adult tree less than 50 per ha. Population with PDI of 1.141 and high density of adult trees was considered as less disturbed while the population with PDI of 6.52 and low adult tree density was considered as heavily disturbed (Table 1).

**TABLE 1:** Disturbance indicators, density of adult trees within the two study populations of *Acacia senegal*; presence or absence of each disturbance indicator is indicated in the bracket where one (1) means presence and zero (0) means absent.

Indicator	Population	
	Heavily disturbed	Less disturbed
Number of cut tree stumps	67% (0.67)	2.1% (0.021)
Number of charcoal kilns	16 (1)	0 (0)
Presence of grazing livestock	Yes (1)	Yes (1)
Level of seedling browsing	85% (0.85)	12% (0.12)
Number of recent settlements	18 (1)	0 (0)
Number of fenced plots	28 (1)	0 (0)
Presence of cultivated land	Yes (1)	0 (0)
Population disturbance index (PDI)	6.52	1.141
Density (Number of adult trees/ha)	22 (008)	414 (061)

*Source*: Omondi et al. (2016).

### 2.2 Sampling of plant material

**FIGURE 1:**
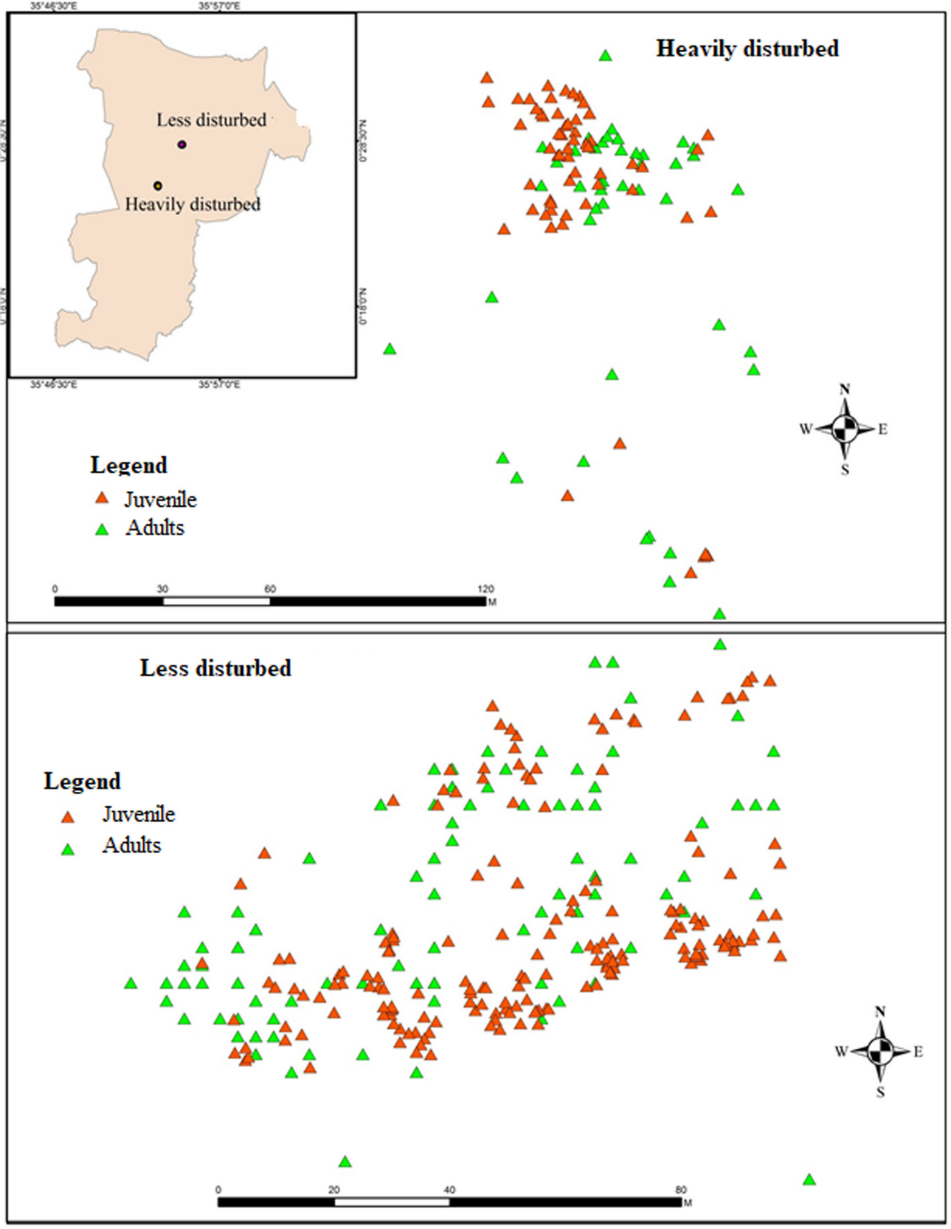
Sampling sites and distribution of sampled individuals (adults and juveniles) within the two populations of *Acacia senegal*.

We investigated two successive generations (adults and juveniles) in each population to estimate the level of realized gene flow within the two populations. Leaf samples from all adults (>5 cm dbh) and juvenile (<5 cm dbh) individuals were collected from a 100 × 200 m quadrat within each population. All the sampled individuals were georeferenced using global positioning system (GPS) and the distributions were as shown in Figure 1. We collected leaf samples from a total of 152 adults and 186 juveniles from the less disturbed population and 83 adults and 59 juveniles from heavily disturbed population.

### 2.3 DNA isolation and microsatellite genotyping

Total genomic DNA was isolated from the leaf samples using a modified cetyltrimethyl ammonium bromide (CTAB) method described by Hanaoka et al. (2013). We performed polymerase chain reaction (PCR) amplifications using 12 polymorphic microsatellite loci developed by Assoumane et al. (2009) and cross amplified in *A. senegal* (Omondi et al., 2010) for all the sampled individuals. The PCR amplification was performed in 10 μL volume containing 10 ng of DNA, 2× Multiplex PCR Master Mix (Qiagen), 0.01 μM fluorescently labeled forward primer, and 0.15 μM reverse primer. PCR conditions were as follows: one cycle at 95°C for 15 min, 67°C for 1.5 min, and 72°C for 1 min, followed by eight cycles at 94°C for 30 s, 65°C for 1.5 min (2°C decrease after every cycle), and 72°C for 1 min, 24 cycles at 94°C for 30 s, 51°C for 1.5 min and 72°C for 1 min, and a single final extension at 60°C for 30 min. Capillary fragment electrophoresis of PCR products were scored against an internal standard (600 Liz size standards) on an ABI 3500 genetic analyser (Applied Biosystems, Califonia, Inc.), and genotype data captured using GeneMapper 5.0 software (Applied Biosystems). We screened 12 microsatellite markers for suitability to perform parentage analysis by testing for conformance to Hardy–Weinberg equilibrium (HWE), presence of null alleles, and linkage disequilibrium. Out of the 12 loci, three showed significant departure from HWE, linkage equilibrium, and presence of high frequency of null alleles. The three loci were discarded and the remaining nine loci were used in the downstream analyses.

### 2.4 SNP discovery and genotyping

We used the Institut de Biologie Intégrative et des Systèmes (IBIS) genotyping by sequencing tool of Université Laval, Quebec, Canada to undertake the genotyping (Torkamaneh et al., 2016). We adapted an in‐house script to identify SNPs (Jérôme Laroch, personal communication). The tags were assigned to individuals by their unique nucleotide barcodes and were trimmed to 64 bp by removing the restriction sites. Restriction site‐associated DNA tags with at least five reads in an individual were included in a sequence alignment across individuals. Qualifying alleles had to be detected in at least half of the sample size (216). To identify loci that were appropriate for the panel, we limited discovery to biallelic SNPs with no more than one polymorphism per 64‐bp sequence. We employed Tassel software version 4.0 (Bradbury et al., 2007) to discover and genotype SNPs from the sequenced tags. Through this, 149,229 SNPs were identified for the 432 *A. senegal* samples.

Out of the 149,229 SNPs identified, we selected a set of 662 SNP markers from the GBS analysis performed on 432 individual tree samples for parentage analysis after further filtering. We set stringent criteria for selecting SNPs as follows: the observed minimum allele frequency (MAF) of 0.3, GC score average > 0.85, and sample call rate > 0.90. In addition, the selected 662 SNPs were further tested for adherence to HWE using a Chi‐square goodness‐of‐fit test in GENEPOP v.4 (Rousset, 2008). This tested significance of the differences between the observed and expected frequencies. SNP markers were included in the analyses only if the *p*‐value of this test was larger than .1. For further screening and filtering, we tested the selected SNP markers for evidence of linkage disequilibrium through correlation of allele frequencies (*r* ^2^ < .11 normally used to measure linkage disequilibrium in unordered SNPs; Pritchard & Przeworski, 2001). The markers were also subjected to assessment of the presence and frequencies of null alleles. Out of the 662 SNPs screened, only 128 passed these tests and were used in downstream analyses. During the filtering process, we also dropped 37 samples that did not have good sequences, most likely due to poor quality of DNA and therefore, 395 samples were finally used in the downstream analyses.

### 2.5 Genetic diversity analysis

The genetic diversity parameters were determined for the two populations and marker types. We determined the following parameters; mean number of alleles (*A*), mean effective number of alleles (*A*_e_), observed heterozygosity (*H*_O_), expected heterozygosity (*H*_E_), and inbreeding coefficient (*F*_IS_) using GenAlEx 6.503 (Peakall & Smouse, 2012).

### 2.6 Parentage analysis

We used the same parentage analysis protocol for both microsatellite and SNP markers using the parentage assignment software, CERVUS version 3.0.7 (Kalinowski et al., 2007). The parentage assignment was evaluated for the most likely parent pairs for all the juveniles from among all possible parents using a likelihood approach. We considered all adult trees of the two populations as candidate parents. The power of our microsatellite and SNP markers to correctly detect putative parents in the populations was estimated by the non‐exclusion probability. We set the software (CERVUS) to assign parentage based on LOD scores. During this analysis, we simulated 100,000 juveniles from parent genotyping data, with a mistyping error rate of 0.001, to estimate the LOD score thresholds for assignment probability. The proportion of parent sampled was held at 80% due to the wide range distribution and potential gene flow patterns of the species. We set a strict confidence level at 95%, while the relaxed confidence was at 80%. Expected heterozygosity (*H*_E_), polymorphism information content (PIC), and non‐exclusion probability (NEP) were calculated for microsatellite and SNP markers. We also evaluated how many SNP loci would be sufficient to ensure assignment of parent pair with 95% confidence. We selected subsets of 128, 100, 80, 60, 40, and 20 most polymorphic SNP loci based on PIC, and repeated simulations using these subsets.

In this study, we assumed that the assigned parent that was physically closest to the seedling was the maternal tree, while we calculated the pollen dispersal distance as the Euclidean distance between the two assigned parent trees. In situations where the same parent was assigned to an offspring as both maternal and paternal parent, we classified the seedling as selfed. However, to avoid the potential biasness of the foregoing assumption, we used all the possible juvenile‐mature combinations to calculate mean seed dispersal distance.

### 2.7 Spatial genetic structure

We undertook the SGS analysis using a combination of microsatellite and GBS‐SNP markers to test the hypothesis of random distribution of *A. senegal* genotypes in space within the two study sites. SGS autocorrelation plots were generated in GenAlex v.6.503 (Peakall & Smouse, 2006, 2012) using Queller and Goodnight's relatedness coefficient (*r*; Queller & Goodnight, 1989). We defined even‐distance classes of 20 m up to the longest distance class within each site. Significance of SGS was calculated by performing 9999 permutations of the geographic locations of individuals to obtain the 95% confidence interval bars. We further subjected the tests to pairwise bootstrapping comparisons for each distance class to obtain the 95% confidence bounds around mean *r*‐values (9999 permutations). We also performed heterogeneity test to assess difference in SGS patterns between the two sites (Smouse et al., 2008). A comparative analysis of spatial structure was done by undertaking Moran's Identity test (Moran's *I*) with 10,000 permutations in SPAGeDi software version 1.5 (Hardy & Vekemans, 2002).

## 3 RESULTS

### 3.1 Genetic diversity

Using both microsatellite and SNP markers, higher genetic diversity (*H*_E_ and number of effective alleles) was found in the juvenile cohort than adults in the heavily disturbed population, while there was lower genetic diversity in juvenile in the less disturbed population. However, fixation index was higher for the juvenile than adult individuals in both populations (Table 2).

**TABLE 2:** Genetic diversity indices for nine microsatellite and 120 SNP markers studied for adults and juveniles in two populations of *Acacia senegal*.

	Heavily disturbed population				Less disturbed population			
	Juveniles		Adults		Juveniles		Adults	
Sample size	56		80		114		148	
Marker type	SNP	Micro	SNP	Micro	SNP	Micro	SNP	Micro
*N*_e_	1.824	5.35	1.480	5.01	1.912	6.34	1.985	6.71
*H*_O_	0.414	0.756	0.272	0.765	0.478	0.742	0.483	0.755
*H*_E_	0.451	0.818	0.318	0.797	0.476	0.839	0.496	0.848
*F*	0.083	0.067	0.155	0.033	−0.0004	0.111	0.026	0.101

Abbreviations: *F*, fixation index; *H*_E_, expected heterozygosity; *H*_O_, observed heterozygosity; Micro, microsatellite; *N*_e_, mean number of effective alleles.

### 3.2 Comparison of marker characteristics between SNPs and microsatellites

Mean number of alleles per locus was 14.7 in the less disturbed population and 13.5 in the heavily disturbed population (overall range 6–17) for microsatellites and two for SNPs in both populations (Table 3). The mean observed and expected heterozygosity, and PIC were greater for microsatellite compared to SNPs. Similarly, non‐exclusion probabilities were lower for SNPs compared to microsatellites (Table 3). The combined marker types showed the lowest values for non‐exclusion probabilities.

**TABLE 3:** Comparison of marker characteristics for SNPs, microsatellite, and a combination of both SNPs and microsatellite markers studied for adult cohorts in two populations of *Acacia senegal*.

Parameter	Less disturbed population			Heavily disturbed population		
	SNPs	Micro	SNPs + micro	SNPs	Micro	SNPs + micro
Number of adult individuals	148	148	148	80	80	80
Number of loci	128	9	137	128	9	137
Mean number of alleles per locus	2	14.7	3.152	2	13.5	2.91
Mean proportion of loci typed	0.997	1.0	0.997	0.998	1.0	0.991
Mean *H*_E_	0.443	0.848	0.529	0.315	0.797	0.365
Mean *H*_O_	0.483	0.755	0.508	0.303	0.765	0.314
Mean PIC	0.342	0.828	0.414	0.257	0.769	0.314
NEP (first parent)	1.4 × 10^−6^	8.858 × 10^−4^	6.15 × 10^−9^	5.85 × 10^−4^	5.05 × 10^−3^	2.30 × 10^−5^
NEP (second parent)	3.55 × 10^−11^	1.729 × 10^−5^	1.48 × 10^−13^	2.0 × 10^−8^	1.65 × 10^−6^	2.15 × 10^−10^
NEP (parent pair)	1.37 × 10^−17^	7.23 × 10^−9^	1.04 × 10^−21^	1.09 × 10^−13^	3.4 × 10^−7^	3.75 × 10^−17^
Assignment at 95% confidence	76.3%	69.1%	79.0%	71.7%	65.8%	86.0%

Abbreviations: *H*_E_, mean expected heterozygosity; *H*_O_, observed heterozygosity; micro, microsatellite; NEP, non‐exclusion probabilities; PIC, polymorphic information content; SNPs, single nucleotide polymorphism.

### 3.3 Parentage analysis and assignments

By using the 128 SNPs, CERVUS assigned 76.3% (87) and 71.7% (38) of the juveniles in the less disturbed and heavily disturbed populations, respectively, to at least one parent with 95% confidence (Table 3). When we used a relaxed confidence level (80%), 99% and 100% of the juvenile in the less disturbed and heavily disturbed populations, respectively, were assigned to at least one parent. Only two juveniles could not be assigned to any putative parent in the less disturbed population due to fewer genotyped loci than the minimum number of loci set during simulation. We used similar probabilities for the nine microsatellites loci; however, only 69.1% and 65.8% of the juveniles in the less disturbed and heavily disturbed populations, respectively, were assigned to at least one parent at 95% confidence level. At a relaxed confidence level (80%), 80% of juveniles in the heavily disturbed population were assigned at least one parent while all (100%) of the juveniles in the less disturbed population were assigned to at least one parent. A combination of the marker types improved the assignment rates in the two populations. Using this combination, CERVUS could assign 79% and 86% juveniles to at least one parent with 95% confidence in the less disturbed and heavily disturbed populations, respectively. At the same confidence level, the combined marker types assigned 56.1% and 60.4% of the juveniles in the less disturbed and heavily disturbed populations, respectively, to a parent pair (two putative parents). While comparing the parent pairs assigned to each juvenile, only 80% of all the assignments in both populations by microsatellite were similar to those assigned by SNPs. We observed almost the same amount (82%) of comparison of assignments in the combined marker types with SNPs. In the entire analysis, CERVUS assigned one parent twice to one juvenile as both the maternal and paternal parent in three occasions in the heavily disturbed population.

### 3.4 Comparison of parentage assignments from different numbers of SNPs

**FIGURE 2:**
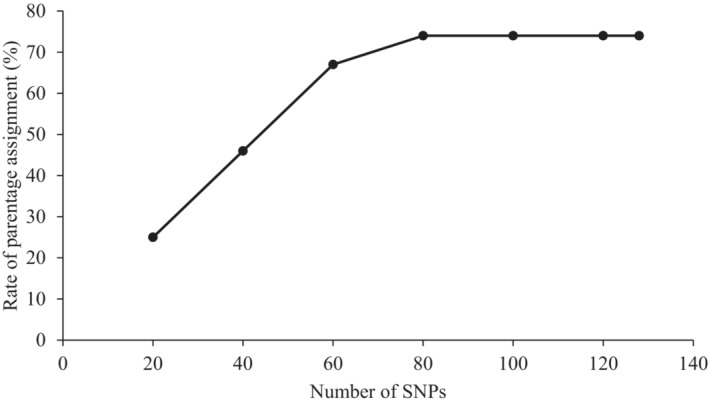
Percentage of CERVUS parentage assignments made using different numbers of the most heterozygous single nucleotide polymorphism (SNPs).

In this comparison, we undertook simulation of parentage assignment with 128 SNPs and with a successive smaller subset of loci down to a minimum of 20 using PIC of the loci. The mean PIC ranged from 0.337 for the whole data set to 0.372 among the 20 most heterozygous loci. Based on this test, the power of assignments of these sets increased with the number of loci until 60 SNPs then reached a plateau at 80 SNPs (Figure 2). Therefore, selecting at least 80 SNP loci would be needed to ensure that parentage would always be assigned with 95% confidence.

### 3.5 Assessment of pollen and seed dispersal

**FIGURE 3:**
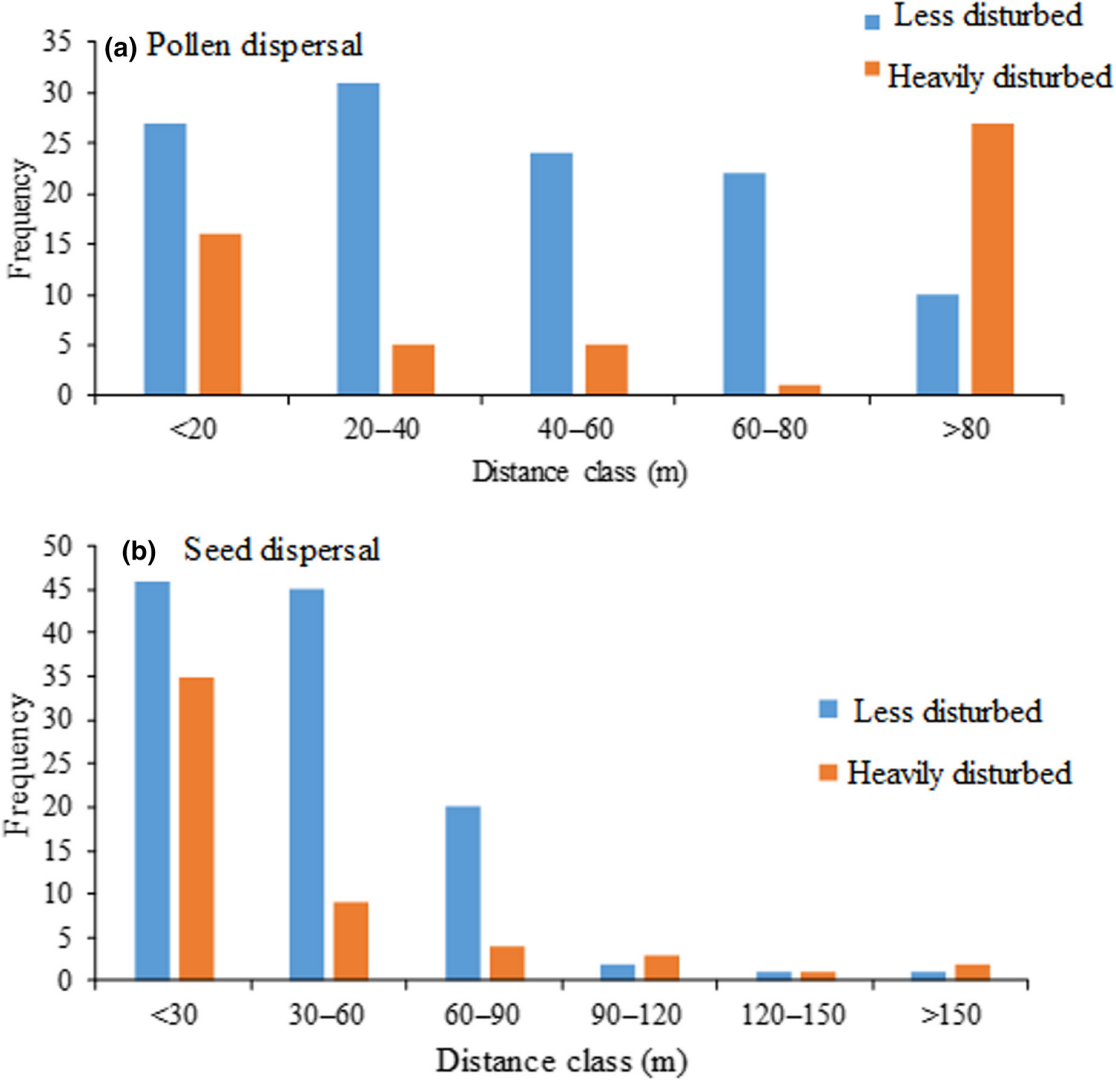
Frequency of pollen and seed dispersal events in different distance classes in *Acacia senegal* in the heavily disturbed (*n* = 53) and less disturbed (*n* = 114) populations.

In this analysis, a combination of SNP and microsatellite markers were utilized. The gene flow within the species spanned distances ranging from 2.1 to 208.6 m (distance from the juvenile to one of the assigned parents or between putative parents). In these assignments, we regarded the closest parent assigned to an offspring as the maternal parent. In this case, *A. senegal* seed movement in the less disturbed population ranged from 2.1 m to about 96 m with a mean seed dispersal distance of 39 m (Table S1). However, seed dispersal beyond 70 m within this population was rare (9.6%). In the heavily disturbed population, seed dispersal distance ranged from 5.1 to 171.2 m with a mean dispersal distance of 38.1 m. A summary of frequency of assignment of maternal parents to a juvenile at different distance classes is as shown in Figure 3. In the case of pollen, we recorded up to 120 and 208.6 m pollen dispersal distances in the less disturbed and heavily disturbed populations, respectively. The mean pollen dispersal distance determined for the less disturbed and heavily disturbed populations was 43.8 and 89.9 m, respectively (Table S2). In the less disturbed population, we found greater proportion (72%) of pollen dispersal frequencies at distances less than 60 m; however, about 52% of pollen dispersal in the heavily disturbed population was more than 80 m.

### 3.6 Spatial genetic structure

**FIGURE 4:**
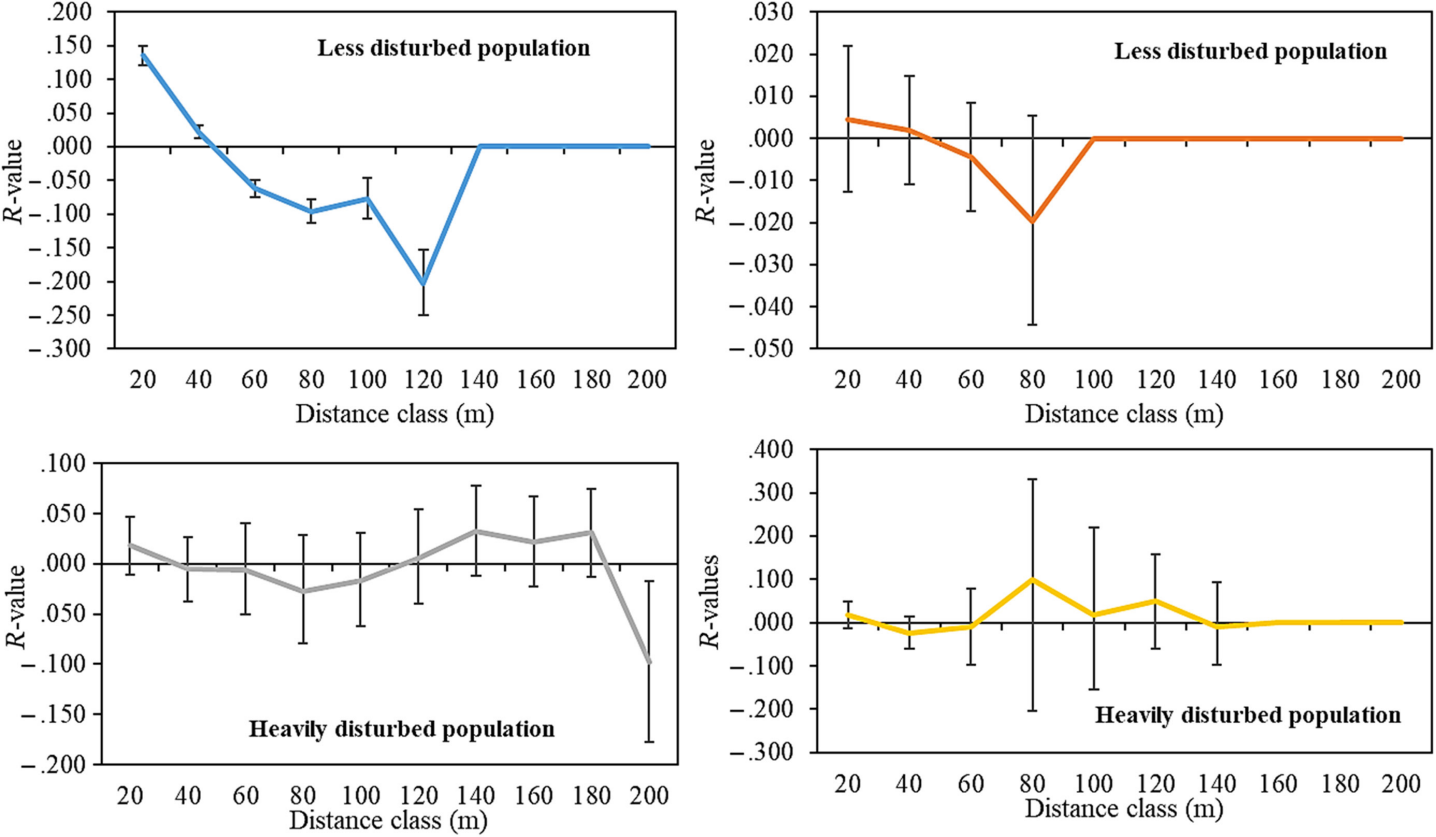
Overlaid SGS autocorrelation plots for up to 200 m, even‐distance classes in 20 m interval, for less disturbed population (adults = 148, juveniles = 114) and heavily disturbed population (adults = 80, juveniles = 53); *R*‐values, average pairwise relatedness among individuals in the shortest distance class. Error bars represents the upper and lower 95% confidence intervals (CIs) around *R*‐values estimated by bootstrap resampling.

To assess the presence of SGS within the cohorts, we divided the spatial distances into 20 m distance classes and a combination of SNP and microsatellite markers were used. Among all the groups analyzed, only the adult cohort of the less disturbed population showed significant spatial structure in all distance classes (*p* < .05; Figure 4). However, we found mean positive values only for the first two distance classes (0–20 and 20–40 m). The mean relatedness (*r*) values for the less disturbed population adult cohort ranged from 0.136 to −0.203. We identified similar significant spatial structure with Moran Moran's *I* in SPAGeDi for the distance classes less than 40 m; however, higher Moran's *I* of 0.153 was found at 20 m distance class (20 m, Moran's *I* = 0.153; 30 m, Moran's *I* = 0.036; 40 m, Moran's *I* = 0.02; for all these distance classes *p* < .05). The heavily disturbed population also showed significant spatial structure in the adult cohort at distance class 200 m (*r* = −.097, *p* = .03), however, no spatial structure was detected in any distance class in this cohort using Moran's *I* analysis. The correlogram of the spatial structure analysis for all the populations and cohorts based on autocorrelation are as shown in Figure 4. The SGS of both the less disturbed and heavily disturbed populations juvenile cohorts were not significant in all the classes (*p* > .05).

**FIGURE 5:**
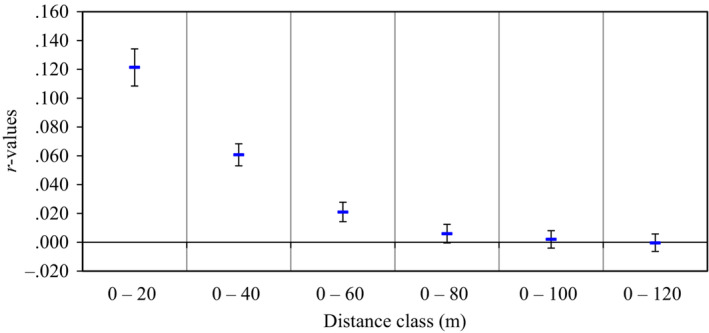
Combined *r*‐values of the adult cohort at multiple distance class sizes for the two study sites.

When comparing the two study populations using heterogeneity tests, we found positive significant difference (*p* < .05) in spatial population structure (heterogeneous spatial structure) at all distance class sizes for adult cohort (Figure 5), however, no significance was observed for the juvenile cohort.

## 4 DISCUSSION

### 4.1 Marker type and data quality

In trying to understand the different maker characteristics, we generated three different sets of genotypic data for the same set of putative parents and progenies using 128 SNPs, nine nuclear microsatellites and a combination of SNPs and microsatellite marker types. The data sets varied in call rate, mistyping and ability to assign parents to offspring. A similar comparative study on *Eucalyptus nitens* using a SNP chip and two sets of microsatellite panels revealed the same results as the one reported in the present study (Telfer et al., 2015). In their work, Telfer et al. (2015) suggested that data quality is the main contributing factor to the difference. For instance, the near Hardy–Weinberg disequilibrium in some of the microsatellite loci observed in the present study could imply that genotypes scored as homozygous could be heterozygous for null alleles, and that one would most likely be observing true null alleles rather than failed amplifications. Such challenges are common mainly when multiplex PCR analyses are performed. This introduces variable amplification efficiencies for some alleles or loci leading to low signal intensity fragment peaks that do not reach the minimum threshold to call an allele. Genotyping errors have been reported to significantly affect the power of a molecular marker; however, low error rates have been reported for SNPs than microsatellite markers as found in the present study (Hauser et al., 2011; Kaiser et al., 2017; Kalinowski et al., 2007; Labuschagne et al., 2015; Walling et al., 2010). As pointed out by Labuschagne et al. (2015), each locus adds linearly to the multilocus error rate and therefore the optimum number of SNP loci to be used should be determined prior to the actual analysis. Based on our analysis, 60 informative SNPs with high PIC and *H*_E_ were as good as 128 SNPs in parentage assignment. Large sets of SNPs appear to provide more information than microsatellite loci; however, a combination of the two marker types improved slightly the assignments of the parents to the offspring in our study. Despite the power of SNPs, microsatellites still have clear benefit over SNPs when the amount of information that can be gathered from a single locus is considered and the fact that fewer loci are required to perform parentage assignment (Telfer et al., 2015). Caution, however, needs to be taken when fewer loci are genotyped, because in such cases an undetected null allele will have a significant impact on the exclusionary power of the marker panel (Labuschagne et al., 2015).

### 4.2 Pollen and seed dispersal

Despite earlier studies suggesting that *A. senegal* predominantly has long‐distance pollen and seed dispersal, we have managed to reveal that the species can also exhibit short‐distance gene flow. However, none of the earlier studies employed parentage analysis as in the present study. Our study showed that majority of gene flow was occurring within the populations; nonetheless, this was happening at different scales in the two populations. Since about 80% of the juveniles were assigned to at least one parent in the two populations, our results suggest that about 20% of the juveniles were realized form seed immigration from outside the two populations. Our finding supports the predominant wind dispersal mechanism of the species that promotes seed dispersal within the population (Bunney et al., 2017). The study revealed higher proportion of seed immigration in the less disturbed than heavily disturbed population. This difference could be attributed to the closeness of the less disturbed population to other populations of *A. senegal* to the north that could act as source of immigrant seed. Generally, mature seeds of *A. senegal* dehisce from the dry pod during dry and windy days and fall sometimes on the ground close to the mother‐tree leading to regeneration close to the maternal tree (Orwa et al., 2009). Nevertheless, some seeds that remain stuck on the pods are blown far away from the maternal tree, supporting migration or immigration among closely located populations. Seed immigration between far populations could also be probable due to the involvement of domestic ungulates and pastoral nomadism which is common within the study area (Andersen et al., 2016). It is documented that small livestock, mainly goats and sheep that are commonly found grazing within the study area, feed on dry pods together with seeds of *A. senegal* and move them from one location to another (Fagg & Allison, 2004). The animals ingest the seeds and disperse them through excretion. Furthermore, during the dry seasons when the seed are ready for dispersal, there are no agricultural crops on the farmlands and the livestock freely move among populations of *A. senegal* in search of pasture and water providing opportunities for migration/immigration and long‐distance seed dispersal (Andersen et al., 2016; Bunney et al., 2017; Omondi et al., 2016). Successful animal ingestion and seed dispersal of several other *Acacia* species have been reported (Stone et al., 2003).

Our study has shown that large proportion of the juveniles in the two populations had a local pollen source suggesting that close to 40% of the pollen actually immigrated from adjacent or far off populations. This rate of pollen immigration was higher than most pollen dispersal so far reported for other African *Acacia* species. Generally, life history traits, for example, large tree size and longevity tend to encourage large amounts of pollen production and extensive dispersal (Millar et al., 2014). In fact, most African *Acacia* species such as *A. senegal* do not have large amounts of floral nectaries that may favor pollen dispersal by birds (Millar et al., 2014). As a result, insects such as ants, moths, wasps, beetles and bees that are known as generalist pollinators, are thought to be the pollen dispersers in most *Acacia* species (González‐Robles et al., 2021; Stone et al., 2003). These insects can influence the tail of dispersal curves and the pollen dispersal directly or through pollen carryover when moving across habitat matrices among populations (Gamba & Muchhala, 2023; Lander et al., 2010).

*Acacia senegal* trees are mainly shrubby and of small stature, but in some cases, they grow up to 15 m high (Raddad et al., 2005). The species also has a prolific flowering tendency and produces small amounts of nectar and pollen that are foraged by bees, which are capable of expanding pollen distribution to several kilometers while foraging across populations (Fagg & Allison, 2004; Harich et al., 2016). The foraging behavior of honeybees indicate that they visit many flowers in one tree then move to nearby trees and finally fly far away to other trees, perhaps a different population, enhancing pollen migration/immigration (Sujii et al., 2021). The abundance of pollen also influences the distance that can be traveled by a honeybee. For instance, during periods of pollen scarcity, the bees may move longer distances in search of pollen and nectar (Pasquet et al., 2008; Peters et al., 2022; Steffan‐Dewenter & Kuhn, 2003). Large proportions of pollen immigration in the two *A. senegal* populations is in fact consistent with the general patterns observed in many studies on common and wide spread tropical tree species that are bee‐pollinated (Byrne et al., 2008; Dick et al., 2003, 2008; Millar et al., 2014; Sebbenn et al., 2011; Wandrag et al., 2017; Ward et al., 2005).

### 4.3 Seed dispersal distance

In both studied populations, we found a strong negative relationship between the probability of maternity and the distance between a seedling and a putative mother tree, as might be expected given barochory as a dominant mode of seed dispersal in Acacias (Tybirk, 1997). The within‐neighborhood average distance between a putative mother tree and a juvenile was short for both the less disturbed and the heavily disturbed populations. This therefore suggests that the majority of seedlings grew close to the mother tree. Although in the case of heavily disturbed population, there was a greater proportion of long‐distance seed dispersal. The seed dispersal patterns were different for the two populations. For instance, there was more frequent short‐distance seed dispersal in the less disturbed population than in the heavily disturbed population. This may be explained by the contrasting density of the two populations as was also suggested for *Boswellia papyrifera* by Bekele (2016). In the present study, seed dispersal is more constrained in the less disturbed than the heavily disturbed population. However, this may only be true, unlike in our case, when considering wind as the predominant agent of dispersal and that density acts as a barrier to seed movement while sparse or open structure allows the pods to be blown to long distances (Curtu et al., 2015). Similar results have been reported for some dryland species such as *Swietenia humilis* (White et al., 2002), however, when animal dispersal is also involved, the effect of population density may not be distinct. *Acacia senegal* is dispersed by both wind and ungulates, however, the activities of animals within the two populations were minimal during the study period, suggesting that the frequent short‐distance seed dispersal found here may have been impacted by tree density as reported for other species (e.g. *Chamaecrista fasciculata*, Fenster, 1991; *Heliconia acuminata*, Cortes et al., 2013). Furthermore, most of the community members living around the two population have abandoned the pastoralism lifestyle and adopted more sedentary lifestyle with some level of peasantry farming. This change of lifestyle has limited free livestock movement among populations, probably affecting *A. senegal* seed dispersal. Constrained seed dispersal was reported for *A. tortilis*, a species that heavily depends on animal dispersal for its seed, in the Negev desert (Ward & Rohner, 1997), although Bekele (2016) suggested animal dispersal as the main contributing factor to the relatively high gene flow among populations of *Boswellia papyrifera* in the drylands of Ethiopia corroborating the theory that free animal movement enhances seed dispersal.

### 4.4 Pollen dispersal distance

The relationship between distance and probability of mating was significant in both study populations. The average distance between mates within the same area was different between the two population. Although pollen flow may reveal a certain level of stochasticity, the greater average distance between mates observed in heavily disturbed population can be partly related to a lower population density that is, larger distances among individuals within the same area and an opened population structure allowing for more extensive pollinator movement within the population (Jones et al., 2022; Peters et al., 2022). The less disturbed population showed dense distribution pattern of *A. senegal* compared to the heavily disturbed population, which may have promoted the frequent short‐distance pollination events in the less disturbed population. This was evidenced by the high frequency of pollination distance of less than 60 m. By contrast, the heavily disturbed population that was characterized by sparse density showed high frequencies of pollination distance greater than 80 m. Though not studied in the present work, our findings are consistent with density‐dependent foraging behavior of bees where a decrease in plant density is accompanied by an increase in flight distance (Byrne et al., 2008; Carper et al., 2022). Similar density dependent pollination distance was reported for *Quercus robur* and *Q. petraea* (Chybicki & Burczyk, 2010). In addition, both the populations were characterized by the presence of other *Acacia* species that may be sharing pollinators (S. F. Omondi, personal observation). Assuming that intraspecific pollination dominates over interspecific pollination, the random distribution of *A. senegal* trees in heavily disturbed population, could have contributed to extensive pollen dispersal than the less disturbed population.

One of the striking results of our study is that no paternal parent was detected for over 40% of the juveniles within the area around the putative mother tree for both populations. This was similar to results reported for other species such as Quercus species (Chybicki & Burczyk, 2010). From our results, pollen mediated gene flow in *A. senegal* appears to follow a bi‐modal pattern, where substantial local pollination is favored, counterbalanced by long‐distance pollen flow (Peters et al., 2022).

### 4.5 Spatial structure

The significant SGS for the adult cohorts in the less disturbed population suggests the occurrence of a historical high frequency of short seed dispersal distances and short‐distance pollination that may have resulted in significant relatedness among individuals at shorter distance classes. However, because there was no difference in the SGS among juvenile cohorts in the two populations, our study suggests that the contemporary combined pollen and seed dispersal dynamics within the two populations are relatively similar. Similar findings were reported for *Koompassia malaccensis* which, like *A. senegal* is insect pollinated and wind dispersed (Noreen et al., 2016). Adult trees in the highly disturbed population showed no SGS while adults in the less disturbed population did, suggesting divergent historical genetic events. Based on allele frequencies, the Moran's *I* test demonstrated that only individuals within the lowest spatial distance were closely related. However, individuals within the medium spatial distance may also be related. Such hypothesis can only be tested with fine spatial scale studies that incorporate more individuals across more spatial distances and from many sites or populations.

Because both maternal and paternal genotypes were unknown, it could not be determined absolutely whether the long‐distance gene flow reported for this study is due to seed or pollen dispersion, however pollen dispersal is the most probable because of the gene dispersal agents involved. Similar long‐distance pollen dispersals (>1000 m) have been reported for other tropical trees pollinated by bees and wasps (Nason & Hamrick, 1997). In fact, paternity studies in angiosperms have revealed that individuals from outside the population frequently pollinate larger portions of the seed crop of a target population (Devlin & Ellstrand, 1990; Gaiotto et al., 2003). It is possible that such long‐distance gene flow events are more frequent than expected in most tropical tree populations.

## 5 CONCLUSION

The study shows clearly that *A. senegal* pollen and seeds can move over long distances and at high rates (Ward et al., 2005). These may be much higher than expected for natural undisturbed populations. Similar results were suggested by Trapnell and Hamrick (2005) on their study of orchid populations. In the present study, *A. senegal* have shown levels of pollen flow that are sufficient to prevent or reduce inbreeding and genetic drift. This suggests that even small fragments of the species population have conservation value as stepping stones that can contribute significantly to maintaining populations' genetic connectivity (Nason & Hamrick, 1997; Sabine et al., 2022; White et al., 2002). Additionally, the primary determinant of pollen flow between isolated trees is pollinator behavior and as pointed out by White et al. (2002), all pollinators have a distance threshold beyond which they cannot travel. In that case, the level to which landscape disturbance affects different plant species will be highly variable (Aldrich & Hamrick, 1998). Although the genetic impacts of anthropogenic disturbances and population fragmentation are undoubtedly complex and will vary among species, this study has clearly demonstrated that remnant of fragmented populations and isolated trees of *A. senegal* will provide a buffer to the deleterious genetic consequences of habitat destruction and may be vital to the future long‐term viability of the species (Salmona et al., 2023).

## AUTHOR CONTRIBUTIONS

**Stephen F. Omondi:** Conceptualization (lead); data curation (equal); formal analysis (lead); funding acquisition (equal); investigation (lead); methodology (equal); project administration (lead); resources (equal); software (lead); supervision (equal); validation (equal); visualization (equal); writing – original draft (lead); writing – review and editing (equal). **Eunice W. Githae:** Conceptualization (supporting); data curation (supporting); formal analysis (supporting); funding acquisition (equal); investigation (supporting); methodology (equal); project administration (supporting); resources (equal); software (supporting); supervision (supporting); validation (supporting); visualization (supporting); writing – original draft (equal); writing – review and editing (equal). **Damase P. Khasa:** Conceptualization (equal); data curation (equal); formal analysis (equal); funding acquisition (equal); investigation (supporting); methodology (supporting); project administration (equal); resources (equal); software (supporting); supervision (equal); validation (equal); visualization (equal); writing – original draft (equal); writing – review and editing (equal).

## CONFLICT OF INTEREST STATEMENT

The authors declare that there is no conflict of interest.

## BENEFITS GENERATED

Benefits from this research accrue from the sharing of our data and results on public databases as described above. A research collaboration was developed with scientists from the three institutions and all collaborators are included as coauthors and the results have been shared broadly, including outside the scientific community. The authors are committed to international scientific partner‐ships, as well as institutional capacity building.

## Supporting information


Appendix S1

## Data Availability

All the data necessary for confirming the conclusions presented in this article is given fully in DRYAD public repository. Dryad, Dataset, https://doi.org/10.5061/dryad.4tmpg4ff6.
