# Supplementary material for: Long‐distance gene flow in Acacia senegal: Hope for disturbed and fragmented populations
Source: Ecol Evol. 2023 Jul 12;13(7):e10292. doi: 10.1002/ece3.10292 (PMC10337015; doi:10.1002/ece3.10292)
Supplement: Supplementary file 1 — Appendix S1 [file ECE3-13-e10292-s001.docx]

**Supplemental Information for:**

**Estimating realized gene flow patterns in the Kenyan population of *Acacia senegal* (syn. *Senegalia senegal*) - an empirical comparison of Single Nucleotide Polymorphic and microsatellite marker variations**

Stephen F. Omondi, Eunice W. Githae, Damase P. Khasa

**Table of Contents:**

| **Table S1.** Geographic distance (m) between the offspring and the assigned parents | Page 1 |
| --- | --- |
| **Table S2.** Geographic distance between the two parents assigned to each offspring | Page 6 |

**Table S1.** Geographic distance (m) between the offspring and the assigned parents

| **Offspring ID** | **First parent ID** | **Distance (m)** | **Second parent ID** | **Distance (m)** |
| --- | --- | --- | --- | --- |
| KMJuv1 | KMAdul43 | 23.6 | KMAdul27 | 38.3 |
| KMJuv10 | KMAdul24 | 7.1 | KMAdul78 | 128.2 |
| KMJuv11 | KMAdul39 | 19.7 | KMAdul14 | 28.2 |
| KMJuv12 | KMAdul54 | 4.0 | KMAdul77 | 159.3 |
| KMJuv13 | KMAdul35 | 15.1 | KMAdul30 | 20.4 |
| KMJuv14 | KMAdul53 | 14.5 | KMAdul84 | 139.2 |
| KMJuv15 | KMAdul54 | 1.1 | KMAdul55 | 6.8 |
| KMJuv16 | KMAdul78 | 131.5 | KMAdul71 | 188.0 |
| KMJuv17 | KMAdul53 | 9.0 | KMAdul39 | 16.0 |
| KMJuv18 | KMAdul1 | 95.3 | KMAdul62 | 123.8 |
| KMJuv19 | KMAdul22 | 26.0 | KMAdul45 | 30.8 |
| KMJuv2 | KMAdul31 | 29.6 | KMAdul30 | 31.8 |
| KMJuv20 | KMAdul46 | 34.3 | KMAdul68 | 149.1 |
| KMJuv21 | KMAdul47 | 30.8 | KMAdul77 | 149.0 |
| KMJuv22 | KMAdul16 | 25.9 | KMAdul59 | 108.5 |
| KMJuv23 | KMAdul53 | 19.7 | KMAdul66 | 101.4 |
| KMJuv24 | KMAdul34 | 7.5 | KMAdul77 | 149.1 |
| KMJuv25 | KMAdul28 | 20.4 | KMAdul44 | 23.2 |
| KMJuv26 | KMAdul10 | 49.9 | KMAdul9 | 51.5 |
| KMJuv27 | KMAdul12 | 22.9 | KMAdul10 | 48.4 |
| KMJuv28 | KMAdul11 | 32.4 | KMAdul10 | 52.7 |
| KMJuv29 | KMAdul11 | 33.4 | KMAdul10 | 53.5 |
| KMJuv3 | KMAdul81 | 94.9 | KMAdul64 | 128.8 |
| KMJuv30 | KMAdul54 | 20.0 | KMAdul77 | 146.9 |
| KMJuv31 | KMAdul47 | 40.5 | KMAdul59 | 105.3 |
| KMJuv32 | KMAdul11 | 11.1 | KMAdul44 | 42.3 |
| KMJuv33 | KMAdul26 | 3.5 | KMAdul64 | 109.6 |
| KMJuv34 | KMAdul28 | 21.2 | KMAdul62 | 119.0 |
| KMJuv35 | KMAdul49 | 46.8 | KMAdul71 | 180.2 |
| KMJuv36 | KMAdul53 | 40.1 | KMAdul1 | 87.6 |
| KMJuv37 | KMAdul10 | 10.3 | KMAdul10 | 10.3 |
| KMJuv38 | KMAdul3 | 60.7 | KMAdul68 | 132.6 |
| KMJuv39 | KMAdul22 | 47.5 | KMAdul9 | 57.4 |
| KMJuv4 | KMAdul46 | 18.6 | KMAdul84 | 156.0 |
| KMJuv40 | KMAdul37 | 22.4 | KMAdul25 | 32.5 |
| KMJuv41 | KMAdul10 | 58.1 | KMAdul79 | 138.8 |
| KMJuv42 | KMAdul81 | 68.2 | KMAdul81 | 141.6 |
| KMJuv43 | KMAdul28 | 38.3 | KMAdul1 | 105.9 |
| KMJuv44 | KMAdul53 | 29.5 | KMAdul80 | 185.2 |
| KMJuv45 | KMAdul53 | 20.3 | KMAdul68 | 161.3 |
| KMJuv47 | KMAdul77 | 167.7 | KMAdul85 | 174.4 |
| KMJuv48 | KMAdul53 | 13.6 | KMAdul13 | 42.0 |
| KMJuv49 | KMAdul42 | 38.0 | KMAdul61 | 134.8 |
| KMJuv5 | KMAdul37 | 17.6 | KMAdul66 | 123.8 |
| KMJuv50 | KMAdul27 | 33.6 | KMAdul1 | 109.0 |
| KMJuv51 | KMAdul37 | 16.5 | KMAdul83 | 130.3 |
| KMJuv52 | KMAdul9 | 69.4 | KMAdul1 | 104.3 |
| KMJuv53 | KMAdul49 | 12.4 | KMAdul27 | 27.5 |
| KMJuv54 | KMAdul18 | 2.5 | KMAdul68 | 148.5 |
| KMJuv55 | KMAdul40 | 20.1 | KMAdul40 | 20.1 |
| KMJuv56 | KMAdul44 | 68.2 | KMAdul54 | 90.1 |
| KMJuv57 | KMAdul61 | 41.3 | KMAdul42 | 74.6 |
| KMJuv6 | KMAdul26 | 18.9 | KMAdul61 | 117.5 |
| KMJuv7 | KMAdul28 | 19.4 | KMAdul22 | 20.5 |
| KMJuv8 | KMAdul4 | 55.4 | KMAdul10 | 63.8 |
| KMJuv9 | KMAdul53 | 15.0 | KMAdul6 | 65.8 |
| LBJuv10 | LBAdul30 | 41.7 | LBAdul127 | 103.2 |
| LBJuv100 | LBAdul67 | 65.3 | LBAdul144 | 92.6 |
| LBJuv101 | LBAdul15 | 54.6 | LBAdul96 | 67.8 |
| LBJuv102 | LBAdul96 | 67.9 | LBAdul145 | 84.1 |
| LBJuv103 | LBAdul83 | 58.5 | LBAdul127 | 102.1 |
| LBJuv104 | LBAdul96 | 64.3 | LBAdul122 | 82.6 |
| LBJuv105 | LBAdul147 | 86.0 | LBAdul112 | 86.8 |
| LBJuv106 | LBAdul55 | 50.4 | LBAdul87 | 51.9 |
| LBJuv107 | LBAdul86 | 52.4 | LBAdul108 | 74.4 |
| LBJuv108 | LBAdul14 | 54.1 | LBAdul126 | 97.1 |
| LBJuv109 | LBAdul44 | 53.1 | LBAdul95 | 62.3 |
| LBJuv110 | LBAdul110 | 84.5 | LBAdul140 | 90.8 |
| LBJuv111 | LBAdul66 | 55.9 | LBAdul95 | 58.7 |
| LBJuv112 | LBAdul37 | 19.4 | LBAdul141 | 89.4 |
| LBJuv113 | LBAdul28 | 14.5 | LBAdul139 | 89.4 |
| LBJuv114 | LBAdul4 | 9.3 | LBAdul138 | 79.9 |
| LBJuv115 | LBAdul28 | 14.3 | LBAdul142 | 76.2 |
| LBJuv116 | LBAdul37 | 18.0 | LBAdul110 | 79.0 |
| LBJuv117 | LBAdul36 | 12.4 | LBAdul25 | 23.5 |
| LBJuv118 | LBAdul146 | 72.9 | LBAdul121 | 74.9 |
| LBJuv12 | LBAdul86 | 41.5 | LBAdul67 | 49.4 |
| LBJuv120 | LBAdul54 | 44.6 | LBAdul123 | 86.6 |
| LBJuv121 | LBAdul81 | 46.3 | LBAdul125 | 85.3 |
| LBJuv122 | LBAdul81 | 47.1 | LBAdul125 | 86.3 |
| LBJuv123 | LBAdul76 | 44.3 | LBAdul117 | 75.6 |
| LBJuv124 | LBAdul71 | 45.5 | LBAdul137 | 75.0 |
| LBJuv125 | LBAdul15 | 41.3 | LBAdul127 | 88.2 |
| LBJuv126 | LBAdul33 | 12.9 | LBAdul19 | 23.7 |
| LBJuv127 | LBAdul15 | 43.0 | LBAdul151 | 68.4 |
| LBJuv128 | LBAdul13 | 32.8 | LBAdul83 | 50.7 |
| LBJuv129 | LBAdul46 | 51.8 | LBAdul122 | 83.1 |
| LBJuv13 | LBAdul142 | 94.4 | LBAdul124 | 101.8 |
| LBJuv130 | LBAdul58 | 49.7 | LBAdul145 | 91.4 |
| LBJuv131 | LBAdul49 | 53.7 | LBAdul114 | 100.7 |
| LBJuv132 | LBAdul53 | 52.4 | LBAdul96 | 82.9 |
| LBJuv133 | LBAdul108 | 78.5 | LBAdul151 | 89.3 |
| LBJuv134 | LBAdul151 | 92.5 | LBAdul121 | 95.0 |
| LBJuv135 | LBAdul108 | 85.6 | LBAdul148 | 107.1 |
| LBJuv136 | LBAdul80 | 70.3 | LBAdul138 | 102.5 |
| LBJuv14 | LBAdul29 | 34.8 | LBAdul66 | 46.8 |
| LBJuv15 | LBAdul75 | 33.9 | LBAdul48 | 46.6 |
| LBJuv16 | LBAdul66 | 36.0 | LBAdul66 | 36.0 |
| LBJuv18 | LBAdul44 | 58.1 | LBAdul138 | 86.2 |
| LBJuv19 | LBAdul66 | 28.5 | LBAdul138 | 82.1 |
| LBJuv2 | LBAdul45 | 55.8 | LBAdul110 | 74.0 |
| LBJuv20 | LBAdul122 | 66.2 | LBAdul137 | 77.7 |
| LBJuv21 | LBAdul27 | 22.7 | LBAdul117 | 57.1 |
| LBJuv22 | LBAdul7 | 39.8 | LBAdul138 | 67.1 |
| LBJuv23 | LBAdul44 | 40.8 | LBAdul135 | 67.8 |
| LBJuv25 | LBAdul22 | 9.8 | LBAdul147 | 67.9 |
| LBJuv26 | LBAdul66 | 14.4 | LBAdul108 | 40.4 |
| LBJuv27 | LBAdul33 | 31.1 | LBAdul30 | 31.2 |
| LBJuv28 | LBAdul4 | 44.2 | LBAdul127 | 75.4 |
| LBJuv29 | LBAdul54 | 14.5 | LBAdul147 | 70.9 |
| LBJuv3 | LBAdul21 | 22.1 | LBAdul126 | 74.2 |
| LBJuv30 | LBAdul57 | 9.3 | LBAdul35 | 28.8 |
| LBJuv31 | LBAdul142 | 44.1 | LBAdul7 | 49.2 |
| LBJuv32 | LBAdul4 | 25.9 | LBAdul56 | 32.2 |
| LBJuv33 | LBAdul142 | 50.6 | LBAdul15 | 27.9 |
| LBJuv34 | LBAdul66 | 34.9 | LBAdul138 | 57.4 |
| LBJuv35 | LBAdul44 | 27.0 | LBAdul78 | 36.2 |
| LBJuv36 | LBAdul92 | 33.2 | LBAdul99 | 38.1 |
| LBJuv37 | LBAdul66 | 32.9 | LBAdul125 | 64.5 |
| LBJuv38 | LBAdul32 | 10.5 | LBAdul103 | 48.0 |
| LBJuv39 | LBAdul30 | 12.0 | LBAdul24 | 28.1 |
| LBJuv4 | LBAdul60 | 34.6 | LBAdul140 | 70.8 |
| LBJuv40 | LBAdul30 | 12.3 | LBAdul121 | 60.4 |
| LBJuv41 | LBAdul70 | 41.2 | LBAdul108 | 56.7 |
| LBJuv42 | LBAdul35 | 3.9 | LBAdul136 | 63.6 |
| LBJuv43 | LBAdul122 | 61.7 | LBAdul110 | 64.6 |
| LBJuv45 | LBAdul36 | 1.5 | LBAdul141 | 72.7 |
| LBJuv46 | LBAdul146 | 58.6 | LBAdul142 | 60.6 |
| LBJuv47 | LBAdul4 | 15.5 | LBAdul56 | 39.9 |
| LBJuv48 | LBAdul110 | 64.7 | LBAdul138 | 65.3 |
| LBJuv49 | LBAdul42 | 30.6 | LBAdul97 | 47.8 |
| LBJuv5 | LBAdul60 | 36.9 | LBAdul10 | 47.9 |
| LBJuv50 | LBAdul31 | 12.0 | LBAdul110 | 61.2 |
| LBJuv51 | LBAdul12 | 43.3 | LBAdul14 | 49.1 |
| LBJuv52 | LBAdul146 | 57.7 | LBAdul119 | 59.4 |
| LBJuv53 | LBAdul20 | 29.1 | LBAdul100 | 48.5 |
| LBJuv54 | LBAdul26 | 15.0 | LBAdul127 | 76.7 |
| LBJuv55 | LBAdul84 | 30.6 | LBAdul122 | 61.8 |
| LBJuv56 | LBAdul31 | 14.1 | LBAdul63 | 43.5 |
| LBJuv57 | LBAdul22 | 42.0 | LBAdul63 | 45.9 |
| LBJuv58 | LBAdul22 | 42.1 | LBAdul117 | 54.9 |
| LBJuv59 | LBAdul103 | 49.0 | LBAdul131 | 64.7 |
| LBJuv6 | LBAdul83 | 17.8 | LBAdul123 | 57.8 |
| LBJuv60 | LBAdul122 | 50.0 | LBAdul7 | 52.3 |
| LBJuv61 | LBAdul108 | 46.3 | LBAdul16 | 47.1 |
| LBJuv62 | LBAdul142 | 42.8 | LBAdul66 | 46.5 |
| LBJuv63 | LBAdul88 | 11.9 | LBAdul102 | 41.6 |
| LBJuv64 | LBAdul142 | 43.6 | LBAdul127 | 56.0 |
| LBJuv65 | LBAdul77 | 41.4 | LBAdul67 | 45.1 |
| LBJuv66 | LBAdul39 | 12.8 | LBAdul35 | 27.8 |
| LBJuv67 | LBAdul15 | 29.6 | LBAdul56 | 42.0 |
| LBJuv68 | LBAdul122 | 39.1 | LBAdul140 | 46.7 |
| LBJuv69 | LBAdul31 | 14.1 | LBAdul121 | 38.4 |
| LBJuv7 | LBAdul95 | 17.3 | LBAdul95 | 17.3 |
| LBJuv71 | LBAdul77 | 35.3 | LBAdul134 | 43.4 |
| LBJuv72 | LBAdul74 | 35.6 | LBAdul147 | 40.7 |
| LBJuv73 | LBAdul18 | 32.7 | LBAdul74 | 35.8 |
| LBJuv74 | LBAdul37 | 19.8 | LBAdul138 | 42.7 |
| LBJuv75 | LBAdul77 | 35.5 | LBAdul98 | 39.2 |
| LBJuv76 | LBAdul81 | 8.1 | LBAdul131 | 50.2 |
| LBJuv77 | LBAdul150 | 35.8 | LBAdul131 | 61.7 |
| LBJuv78 | LBAdul4 | 28.8 | LBAdul127 | 62.9 |
| LBJuv79 | LBAdul55 | 6.2 | LBAdul39 | 35.0 |
| LBJuv8 | LBAdul38 | 45.0 | LBAdul142 | 56.5 |
| LBJuv80 | LBAdul96 | 41.0 | LBAdul140 | 58.3 |
| LBJuv81 | LBAdul37 | 44.4 | LBAdul123 | 54.4 |
| LBJuv82 | LBAdul37 | 41.0 | LBAdul136 | 57.3 |
| LBJuv83 | LBAdul10 | 52.6 | LBAdul136 | 56.0 |
| LBJuv84 | LBAdul142 | 46.3 | LBAdul127 | 57.4 |
| LBJuv85 | LBAdul64 | 8.8 | LBAdul22 | 32.4 |
| LBJuv86 | LBAdul45 | 13.9 | LBAdul38 | 29.8 |
| LBJuv87 | LBAdul88 | 10.6 | LBAdul45 | 13.0 |
| LBJuv88 | LBAdul138 | 31.6 | LBAdul127 | 38.5 |
| LBJuv9 | LBAdul138 | 30.1 | LBAdul127 | 37.1 |
| LBJuv97 | LBAdul44 | 6.9 | LBAdul121 | 23.9 |
| LBJuv98 | LBAdul108 | 20.9 | LBAdul108 | 20.9 |
| LBJuv99 | LBAdul147 | 30.2 | LBAdul130 | 49.7 |

**Table S2.** Geographic distance between the two parents assigned to each offspring

| **First parent ID** | **Second parent ID** | **Distance** |
| --- | --- | --- |
| KMAdul10 | KMAdul10 | 0.0 |
| KMAdul40 | KMAdul40 | 0.0 |
| LBAdul66 | LBAdul66 | 0.0 |
| LBAdul95 | LBAdul95 | 0.0 |
| LBAdul108 | LBAdul108 | 0.0 |
| LBAdul45 | LBAdul88 | 3.1 |
| LBAdul110 | LBAdul122 | 4.4 |
| LBAdul67 | LBAdul77 | 4.4 |
| KMAdul30 | KMAdul31 | 5.6 |
| KMAdul54 | KMAdul55 | 6.7 |
| KMAdul77 | KMAdul85 | 6.8 |
| LBAdul110 | LBAdul140 | 6.9 |
| LBAdul142 | LBAdul146 | 6.9 |
| LBAdul12 | LBAdul14 | 8.7 |
| KMAdul30 | KMAdul35 | 9.2 |
| LBAdul44 | LBAdul95 | 9.3 |
| LBAdul127 | LBAdul138 | 9.8 |
| LBAdul127 | LBAdul138 | 9.8 |
| LBAdul30 | LBAdul33 | 9.8 |
| LBAdul124 | LBAdul142 | 11.1 |
| LBAdul122 | LBAdul140 | 11.1 |
| LBAdul127 | LBAdul142 | 12.7 |
| LBAdul18 | LBAdul74 | 12.7 |
| LBAdul77 | LBAdul98 | 12.7 |
| LBAdul127 | LBAdul142 | 12.7 |
| LBAdul35 | LBAdul39 | 15.4 |
| LBAdul44 | LBAdul78 | 15.4 |
| LBAdul110 | LBAdul138 | 15.4 |
| LBAdul15 | LBAdul56 | 15.4 |
| LBAdul19 | LBAdul33 | 15.8 |
| LBAdul145 | LBAdul96 | 16.6 |
| LBAdul48 | LBAdul75 | 16.6 |
| LBAdul38 | LBAdul45 | 18.8 |
| LBAdul112 | LBAdul147 | 18.8 |
| LBAdul121 | LBAdul146 | 18.8 |
| LBAdul119 | LBAdul146 | 18.8 |
| LBAdul25 | LBAdul36 | 19.5 |
| KMAdul10 | KMAdul11 | 20.3 |
| KMAdul10 | KMAdul11 | 20.3 |
| LBAdul122 | LBAdul96 | 20.7 |
| LBAdul122 | LBAdul137 | 21.6 |
| LBAdul92 | LBAdul99 | 21.6 |
| KMAdul22 | KMAdul28 | 23.8 |
| LBAdul22 | LBAdul63 | 24.7 |
| LBAdul22 | LBAdul64 | 24.7 |
| LBAdul130 | LBAdul147 | 24.9 |
| KMAdul39 | KMAdul53 | 25.0 |
| LBAdul42 | LBAdul97 | 26.4 |
| LBAdul108 | LBAdul66 | 26.6 |
| LBAdul108 | LBAdul70 | 27.6 |
| KMAdul25 | KMAdul37 | 27.9 |
| LBAdul131 | LBAdul150 | 28.0 |
| LBAdul140 | LBAdul96 | 28.0 |
| KMAdul13 | KMAdul53 | 28.4 |
| LBAdul121 | LBAdul151 | 28.5 |
| LBAdul15 | LBAdul96 | 29.1 |
| LBAdul121 | LBAdul44 | 29.3 |
| KMAdul27 | KMAdul43 | 30.4 |
| LBAdul135 | LBAdul44 | 30.4 |
| LBAdul24 | LBAdul30 | 30.4 |
| LBAdul108 | LBAdul86 | 30.6 |
| LBAdul100 | LBAdul20 | 30.9 |
| LBAdul122 | LBAdul46 | 31.5 |
| LBAdul31 | LBAdul63 | 31.8 |
| LBAdul138 | LBAdul44 | 32.3 |
| LBAdul122 | LBAdul84 | 32.3 |
| LBAdul102 | LBAdul88 | 32.3 |
| KMAdul44 | KMAdul54 | 34.0 |
| KMAdul64 | KMAdul81 | 34.3 |
| KMAdul22 | KMAdul45 | 34.5 |
| KMAdul10 | KMAdul12 | 34.8 |
| LBAdul103 | LBAdul131 | 35.0 |
| LBAdul138 | LBAdul80 | 35.2 |
| LBAdul110 | LBAdul45 | 35.2 |
| LBAdul108 | LBAdul151 | 35.2 |
| LBAdul67 | LBAdul86 | 37.1 |
| LBAdul108 | LBAdul148 | 37.2 |
| LBAdul35 | LBAdul57 | 37.7 |
| KMAdul10 | KMAdul9 | 38.2 |
| LBAdul55 | LBAdul87 | 39.1 |
| LBAdul66 | LBAdul95 | 39.1 |
| KMAdul27 | KMAdul49 | 39.4 |
| LBAdul4 | LBAdul56 | 39.6 |
| LBAdul4 | LBAdul56 | 39.6 |
| LBAdul53 | LBAdul96 | 40.2 |
| LBAdul15 | LBAdul151 | 40.2 |
| LBAdul123 | LBAdul83 | 40.3 |
| KMAdul28 | KMAdul44 | 40.5 |
| LBAdul125 | LBAdul81 | 40.6 |
| LBAdul125 | LBAdul81 | 40.6 |
| LBAdul10 | LBAdul60 | 41.2 |
| LBAdul39 | LBAdul55 | 41.2 |
| LBAdul117 | LBAdul76 | 42.9 |
| KMAdul14 | KMAdul39 | 43.2 |
| LBAdul127 | LBAdul83 | 43.7 |
| LBAdul142 | LBAdul15 | 43.9 |
| LBAdul108 | LBAdul16 | 44.2 |
| LBAdul29 | LBAdul66 | 45.6 |
| KMAdul11 | KMAdul44 | 46.3 |
| LBAdul121 | LBAdul31 | 46.3 |
| LBAdul131 | LBAdul81 | 46.4 |
| LBAdul114 | LBAdul49 | 47.2 |
| LBAdul103 | LBAdul32 | 48.8 |
| LBAdul121 | LBAdul30 | 49.5 |
| LBAdul110 | LBAdul31 | 49.5 |
| LBAdul142 | LBAdul38 | 49.5 |
| LBAdul144 | LBAdul67 | 50.3 |
| LBAdul142 | LBAdul66 | 50.7 |
| LBAdul125 | LBAdul66 | 50.7 |
| LBAdul123 | LBAdul54 | 54.0 |
| LBAdul137 | LBAdul71 | 55.0 |
| LBAdul127 | LBAdul15 | 55.7 |
| LBAdul117 | LBAdul27 | 55.7 |
| KMAdul1 | KMAdul62 | 55.7 |
| KMAdul71 | KMAdul78 | 56.5 |
| LBAdul134 | LBAdul77 | 56.8 |
| LBAdul147 | LBAdul54 | 57.0 |
| LBAdul147 | LBAdul74 | 57.5 |
| LBAdul138 | LBAdul66 | 57.5 |
| LBAdul138 | LBAdul66 | 57.5 |
| LBAdul145 | LBAdul58 | 59.0 |
| LBAdul136 | LBAdul37 | 60.0 |
| LBAdul110 | LBAdul37 | 62.1 |
| LBAdul138 | LBAdul37 | 62.5 |
| LBAdul140 | LBAdul60 | 62.6 |
| LBAdul13 | LBAdul83 | 63.6 |
| LBAdul117 | LBAdul22 | 63.7 |
| LBAdul127 | LBAdul30 | 64.5 |
| LBAdul136 | LBAdul35 | 67.5 |
| LBAdul123 | LBAdul37 | 68.0 |
| LBAdul141 | LBAdul37 | 71.1 |
| KMAdul1 | KMAdul9 | 71.4 |
| KMAdul53 | KMAdul6 | 73.7 |
| LBAdul141 | LBAdul36 | 74.1 |
| LBAdul147 | LBAdul22 | 74.4 |
| KMAdul10 | KMAdul4 | 75.2 |
| LBAdul127 | LBAdul26 | 75.2 |
| LBAdul142 | LBAdul28 | 77.2 |
| LBAdul138 | LBAdul4 | 80.2 |
| KMAdul81 | KMAdul81 | 81.0 |
| KMAdul22 | KMAdul9 | 81.2 |
| LBAdul126 | LBAdul21 | 81.6 |
| KMAdul1 | KMAdul27 | 87.2 |
| LBAdul139 | LBAdul28 | 88.5 |
| LBAdul127 | LBAdul4 | 88.5 |
| LBAdul127 | LBAdul4 | 88.5 |
| LBAdul122 | LBAdul7 | 89.2 |
| LBAdul126 | LBAdul14 | 92.7 |
| LBAdul142 | LBAdul7 | 92.9 |
| LBAdul138 | LBAdul7 | 98.2 |
| KMAdul1 | KMAdul28 | 98.9 |
| KMAdul3 | KMAdul68 | 99.3 |
| KMAdul42 | KMAdul61 | 100.5 |
| KMAdul42 | KMAdul61 | 100.5 |
| LBAdul10 | LBAdul136 | 102.0 |
| KMAdul1 | KMAdul53 | 104.3 |
| KMAdul37 | KMAdul66 | 111.7 |
| KMAdul26 | KMAdul64 | 112.8 |
| KMAdul26 | KMAdul61 | 113.9 |
| KMAdul16 | KMAdul59 | 115.8 |
| KMAdul53 | KMAdul66 | 119.1 |
| KMAdul37 | KMAdul83 | 122.3 |
| KMAdul28 | KMAdul62 | 122.9 |
| KMAdul24 | KMAdul78 | 122.9 |
| KMAdul47 | KMAdul59 | 138.2 |
| KMAdul34 | KMAdul77 | 145.7 |
| KMAdul18 | KMAdul68 | 147.6 |
| KMAdul10 | KMAdul79 | 149.6 |
| KMAdul53 | KMAdul84 | 150.1 |
| KMAdul53 | KMAdul68 | 159.0 |
| KMAdul54 | KMAdul77 | 162.6 |
| KMAdul54 | KMAdul77 | 162.6 |
| KMAdul46 | KMAdul84 | 165.4 |
| KMAdul46 | KMAdul68 | 168.2 |
| KMAdul47 | KMAdul77 | 170.5 |
| KMAdul53 | KMAdul80 | 172.4 |
| KMAdul49 | KMAdul71 | 199.4 |
